# Requirement of vasculogenesis and blood circulation in late stages of liver growth in zebrafish

**DOI:** 10.1186/1471-213X-8-84

**Published:** 2008-09-16

**Authors:** Svetlana Korzh, Xiufang Pan, Marta Garcia-Lecea, Cecilia Lanny Winata, Xiaotao Pan, Thorsten Wohland, Vladimir Korzh, Zhiyuan Gong

**Affiliations:** 1 Department of Biological Sciences, National University of Singapore; 2 Institute of Molecular and Cell Biology, Singapore; 3 Department of Chemistry, National University of Singapore

## Abstract

**Background:**

Early events in vertebrate liver development have been the major focus in previous studies, however, late events of liver organogenesis remain poorly understood. Liver vasculogenesis in vertebrates occurs through the interaction of endoderm-derived liver epithelium and mesoderm-derived endothelial cells (ECs). In zebrafish, although it has been found that ECs are not required for liver budding, how and when the spatio-temporal pattern of liver growth is coordinated with ECs remains to be elucidated.

**Results:**

To study the process of liver development and vasculogenesis *in vivo*, a two-color transgenic zebrafish line *Tg(lfabf:dsRed; elaA:EGFP) *was generated and named LiPan for liver-specific expression of DsRed RFP and exocrine pancreas-specific expression of GFP. Using the LiPan line, we first followed the dynamic development of liver from live embryos to adult and showed the formation of three distinct yet connected liver lobes during development. The LiPan line was then crossed with *Tg(fli1:EGFP)*^y1 ^and vascular development in the liver was traced *in vivo*. Liver vasculogenesis started at 55–58 hpf when ECs first surrounded hepatocytes from the liver bud surface and then invaded the liver to form sinusoids and later the vascular network. Using a novel non-invasive and label-free fluorescence correction spectroscopy, we detected blood circulation in the liver starting at ~72 hpf. To analyze the roles of ECs and blood circulation in liver development, both *cloche *mutants (lacking ECs) and Tnnt2 morphants (no blood circulation) were employed. We found that until 70 hpf liver growth and morphogenesis depended on ECs and nascent sinusoids. After 72 hpf, a functional sinusoidal network was essential for continued liver growth. An absence of blood circulation in Tnnt2 morphants caused defects in liver vasculature and small liver.

**Conclusion:**

There are two phases of liver development in zebrafish, budding and growth. In the growth phase, there are three distinct stages: avascular growth between 50–55 hpf, where ECs are not required; endothelium-dependent growth, where ECs or sinusoids are required for liver growth between 55–72 hpf before blood circulation in liver sinusoids; and circulation-dependent growth, where the circulation is essential to maintain vascular network and to support continued liver growth after 72 hpf.

## Background

Hepatogenesis in zebrafish has been intensively studied in recent years and these studies indicate that the early stages are rather similar in mice and zebrafish [1-9]. As detected by expression of *ceruloplasmin *[2] and GFP expression in the gut-GFP transgenic line [5], the liver anlagen initially appears as a small compact protrusion of the intestinal rod at ~30 hpf (hours postfertilization) on the left side of the embryo. By 2 dpf (days postfertilization), the liver bud starts to enlarge and forms the hepatic duct connecting the intestinal bulb primordium to the liver, which marks the end of the budding phase and the beginning of the growth phase [5,10]. Despite these progresses, the late events of hepatogenesis and the underlying developmental mechanisms are not fully understood. A further analysis of liver development during the growth period will complement existing studies and will be highly desirable as during this period the liver develops its vasculature and becomes functional.

Liver organogenesis in vertebrates coincides with the appearance of ECs adjacent to the endoderm. In mice, before the formation of functional blood vessels, ECs provide a very early morphogenetic signal to the liver bud. It has been shown that in *Vegfr2/Flk-1*^-/- ^mouse embryos that lack ECs, the liver undergoes the initial step of hepatic specification and forms a multi-layered epithelium anlage, but further liver morphogenesis fails prior to mesenchyme invasion [11]. In zebrafish *cloche *mutant embryos in which EC differentiation is disrupted at the stage prior to *Vegfr2/Flk-1 *expression [12,13], liver budding and differentiation have been found to proceed normally [5]. However, the interaction between vasculature and liver, as well as the role of ECs and growing vessels in late hepatogenesis in zebrafish, remain largely unclear.

To investigate the late events of liver development and the roles of vasculogenesis and blood flow, in the present study, a two-color transgenic line *Tg(lfabf:ds-Red; elaA:EGFP) *named LiPan for Liver- and Pancreas-specific transgene expression, was generated and it displayed strong expression of Ds-Red RFP (red fluorescent protein) in the liver and GFP in the exocrine pancreas. To visualize liver vasculogenesis, the LiPan line was crossed with *Tg(fli1:EGFP)*^y1^[14]. Liver vasculogenesis and growth was traced in embryos of compound transgenics on wild type and mutant backgrounds using single- and multi-photon laser scanning microscopy. By taking the advantage of recently developed non-invasive method of fluorescence correlation spectroscopy (FCS) [15], the initiation of blood flow in liver vessels in live zebrafish embryos was determined. Finally, by analyses of the EC-less *cloche *mutant and blood flow defective Tnnt2 morphants, we demonstrated the interaction of ECs with hepatocytes and blood flow act in sequence to sustain the continued growth of liver in late development.

## Results

### Liver morphogenesis in two-color LiPan transgenic line

**Figure 1:**
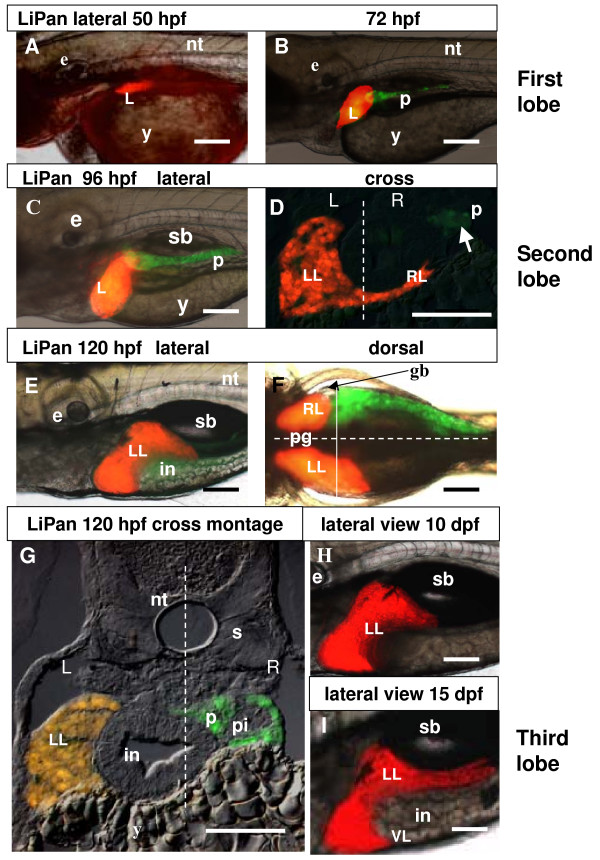
**Liver development in LiPan transgenic zebrafish**. (A, B) Initial RFP expression in the liver starts at 48–53 hpf (A) and GFP expression in the exocrine pancreas starts at 67–72 hpf (B). (C) Expression of RFP and GFP at 96 hpf. (D) Cross section of a 96 hpf larvae shows RFP-positive liver. GFP-expressing exocrine pancreas is faintly visible (arrow). The dotted line represents the midline of the larva and left (L) and right (R) sides are indicated. (E, F) Expression of RFP and GFP in the larva at 120 hpf: lateral (E) and dorsal view (F). The dotted horizontal line in (F) represents the midline with the right side at the top. The solid vertical line represents the plan of the cross section in (G). (G) A cross section to illustrate morphology of internal organs and expression of transgenes right (R) sides are indicated. (H, I) Lateral view of RFP-expressing liver at 10 dpf (H) and in a 120-hpf larva. The dotted line represents the midline of the larva and left (L) and 15dpf (I). Abbreviations: e, eye; gb, gall bladder; in, intestine; L, liver; LL, left lobe; RL, right lobe; nt, notochord; p, pancreas; pi, principal islet; pg, pigment; s, somite, VL, ventral lobe. In all whole mount images anterior is towards the left. Scale bars, 125 μm.

The two-color LiPan transgenic line was generated by co-injection of the dsRed RFP reporter gene under a liver-specific *lfabp *promoter [16] and the GFP reporter gene under an exocrine pancreas-specific *elaA *promoter [17]. The LiPan transgenic line allowed simultaneous monitoring of the temporal and spatial development of two major digestive organs in live embryos/fish, the RFP-expressing liver and GFP-expressing exocrine pancreas. Generally, the red fluorescence appeared in the position of the liver bud from the left side at 48–53 hpf (Figure 1A) and the green fluorescence appeared in position of the exocrine pancreas at the right side of the dorso-anterior intestine of the embryos at 67–72 hpf with its anterior part (head pancreas) at the level of the 3^rd ^somite and its posterior part (tail pancreas) at the level of the 6^th ^somite. At 72 hpf, the liver actively grew and further expanded laterally and antero-ventrally beyond the 1^st ^somite (the first or left lobe, Figure 1B), it remained its initial shape and was restricted to the left side of the body. At 80–84 hpf, the liver rapidly expanded in several directions: anteriorly towards the ear vesicle and ventrally across the midline to the right side of the body (Figure 1C), where it formed the second lobe (the right or gall-bladder lobe, Figure 1D). Soon after, hepatocytes of the right lobe established tight contact with the gall bladder (80–96 hpf), and a yellow-greenish substance, probably bile, appeared in the intestinal bulb (not shown), suggesting that at least some hepatocytes were sufficiently mature to function in food digestion.

At 120 hpf, the left lobe spread even more anteriorly to the mid-ear and posteriorly along the gut (Figure 1E). While anteriorly both lobes were almost at the same A-P level and in contact with the pericardial cavity, posteriorly the right lobe was much shorter due to the presence of the gall bladder and pancreas immediately posterior to it (Figure 1F). The size of both liver and exocrine pancreas varied slightly, but at this A-P level the liver and pancreas were invariably projected onto opposite sides of the body as compact and separated organs (Figure 1D, F, G).

**Figure 2:**
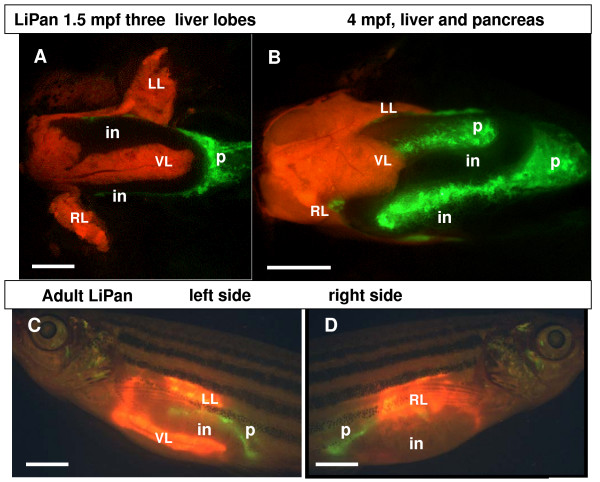
**Liver and pancreas in adult LiPan fish**. (A, B) Ventral view of the three liver lobes (red) in the dissected LiPan zebrafish at 1.5 mpf (A) and 4 mpf (B). The three lobes of liver are indicated by LL, left lobe; RL, right lobe; and VL, ventral lobe. The exocrine pancreas (p) is in green. (C, D) lateral view of LiPan adult: the left side (C) and the right side (D). Other abbreviation: in, intestine. Scale bars, 1500 μm.

Between 5–10 dpf, the liver continued to grow in size. Around 15 dpf, the ventral most portion of the liver began expanding posteriorly (Figure 1H, I), leading to the formation of the third flat ventral lobe caudally to the first two lobes. The timing of development of the third lobe varied between 15–20 dpf. After 15 dpf, the exocrine pancreas was enlarged mostly posteriorly following the looped intestine [17]. We observed the exocrine pancreas in the adult zebrafish to be a less compact structure compared to that in the larvae. GFP-expressing exocrine tissue was consistently observed only on one side of the intestinal mesentery along all three intestinal loops and it never surrounded the intestine (21 male and female LiPan zebrafish, Figure 2A, B), [17]. While the liver in general occupied the anterior region of the body cavity, exocrine pancreas occupied the posterior part of the body cavity (Figure 2B). In addition, liver-expressed RFP and exocrine pancreas-expressed GFP could be observed in live adult LiPan zebrafish under a fluorescent microscope (Figure 2C, D).

### Formation of sinusoidal network in the zebrafish liver

**Figure 3:**
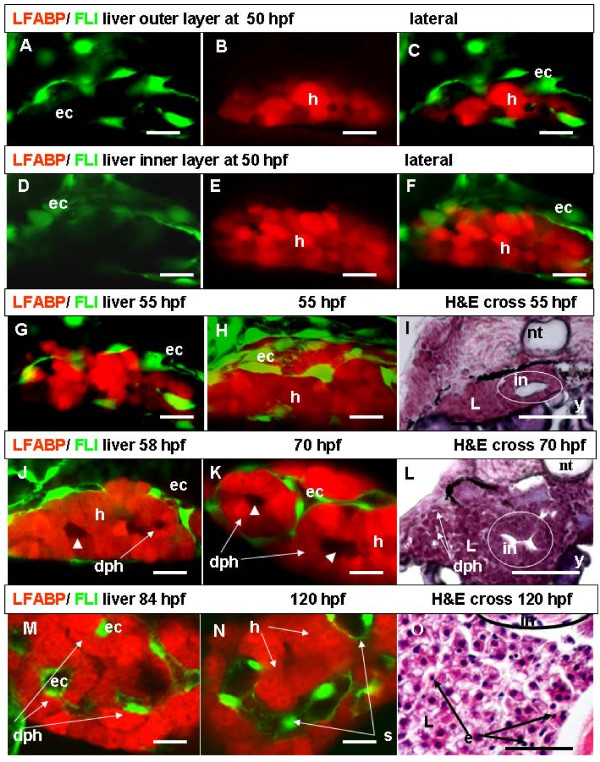
**Liver vasculogenesis**. (A-C) Left-lateral confocal live images of liver of LiPan/*Tg(fli1:EGFP)*^y1 ^larva at 50 hpf. Discrete ECs frame outer layers of the liver bud (A-C) while no ECs are present in the inner layer (D-F). Panels A and D are images under a GFP filter, panels B and E under a RFP filter, and panels C and F are combinations of GFP and RFP images. (G, H) Left-lateral confocal live images of LiPan/*Tg(fli1:EGFP)*^y1 ^liver at 55 hpf: external liver layers (G) and all liver layers (H). ECs establish contact and enter superficial hepatocytes layer. (I) Hematoxylin and eosin (H&E) staining of a cross section of an embryo at 55 hpf to show tightly packed hepatocytes inside the liver. (J, K, M, N) Confocal sections of internal liver layers at 58 hpf (J), 70 hpf (K), 84 hpf (M) and 120 hpf (N). At 58 hpf, RFP negative areas in the innermost liver layers (arrowhead) and a daisy pattern of hepatocytes are formed around this areas (J). At 70 hpf, the daisy pattern of hepatocytes around RFP negative areas extended to the superficial layers and ECs surround the daisy clusters of hepatocytes from outside (K). In some cases, ECs are inside of daisy clusters of hepatocytes (M). By 120 hpf, the size of sinusoids significantly increases (N). (L, O) H&E staining of cross sections of embryos at 70 hpf (L) and 120 hpf (O). Note a daisy pattern of hepatocytes in (L) and erythrocytes with pink cytoplasm in liver sinusoids (O). Abbreviations: dph, daisy pattern of hepatocytes; ec, endothelial cells; e, erythrocyte; h, hepatocyte; in, intestine; nt, notochord; L, liver; s, sinusoid; y, yolk. In all whole mount images anterior is towards the left. Scale bars represent 625 μm except for Panels (I, L, O), where the scale bars are 125 μm.

To observe *in vivo *how and when the ECs developed the liver vascular network, we crossed the LiPan line with the transgenic line *Tg(fli1:EGFP)*^y1 ^in which GFP was specifically expressed in ECs [14]. In LiPan/*Tg(fli1:EGFP)*^y1 ^embryos, we directly observed events of liver vasculogenesis from the late liver budding stage and during the liver growth phase. After the liver budding stage was complete at 50 hpf, distinct GFP-expressing ECs bordered the liver. These ECs probably represented components of nascent branches from the subintestinal vessels [5,18]. At this stage, hepatocytes on the surface and inside the liver bud remained tightly interconnected and showed no obvious morphological organization as observed by confocal microscopy (Figure 3A–F) and histological staining (not shown). During the growth phase at 55–58 hpf, GFP-expressing ECs edged the liver completely and sprouts of ECs contacted seemingly less associated superficial hepatocytes and penetrated between them (Figure 3G, H), whereas inner hepatocytes remained tightly connected (Figure 3H, I).

As sprouts of ECs penetrated between the surface hepatocytes of the liver, some RFP-negative areas appeared between inner layers of hepatocyte and a distinctive daisy pattern of hepatocytes around RFP-negative areas became visible in the innermost liver layers (Figure 3J). Subsequently, ECs penetrate deeper and appeared between internal layers of hepatocytes and RFP-negative areas were extended to near the liver surface layers (Figure 3K). Thus, the timing of ECs and hepatocyte interaction seems correlated with the daisy organization of hepatocytes around the RFP-negative areas. The RFP-negative areas might represent ductal cells previously identified in zebrafish liver at around 60 hpf [19]. While the liver extended laterally and anteriorly, the daisy pattern of hepatocytes was evident in nearly all liver layers and ECs appeared around the daisy patterned hepatocytes (Figure 3K). However, in some cases, we also observed ECs inside the daisy structure of hepatocytes as well (not shown at this time point but see Figure 3M) and it seemed that ECs and hepatocytes moved towards each other. Between 68–72 hpf, ECs formed the structure of the first sinusoids. The sinusoids were 5–8 μm in diameter and contributed to the substantial increase of the liver in size. Up to this stage, ECs were arranged into non-functional vessels as no erythrocytes were detected within the liver (Figure 3I, L).

**Figure 4:**
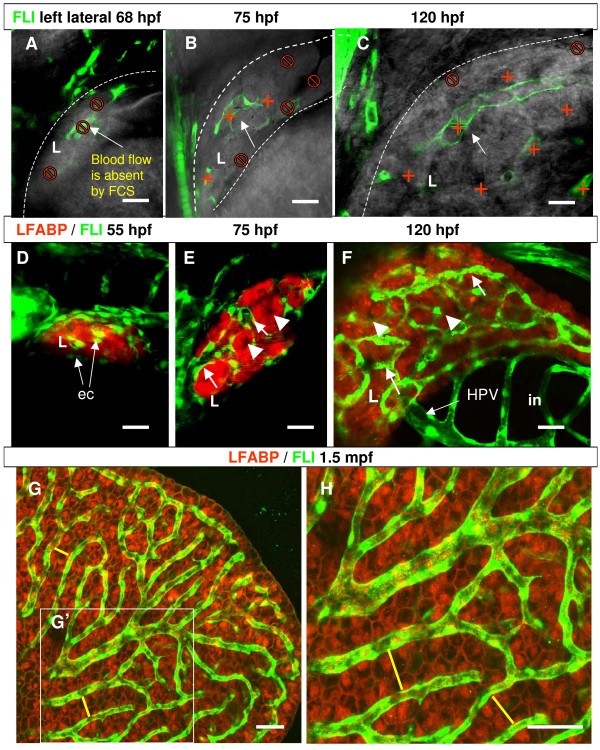
**Assessment of blood circulation in the developing liver of zebrafish**. (A-C) Left lateral 3D confocal sections of liver at the level of sinusoids where blood flow was measured. In the most outer liver layers where the first sinusoids were formed, blood flow was absent at 68 hpf (A). Blood flow was detected in sinusoids of external dorso-lateral part of liver parenchyma at 75 hpf (B) and in all measured sites of liver sinusoids at 120 hpf (C). Red cross, points of measurement at the different focal distance as deep as 70–80 μm from the liver surface; "No" sign, points in sinusoids where no blood flow was detected. The edges of livers are marked by dash lines. Arrows indicate the focused sinusoids for FCS measurement in the picture. (D-F) Confocal projections show three stages of liver vasculogenesis. ECs start to contact the surface layer of hepatocytes in the liver bud at 55 hpf (D), first sinusoids form between external layers of hepatocytes at 75 hpf (E) and have well developed sinusoidal network at 120 hpf (F). Arrows are endothelial cells and sinusoids; arrowheads are clusters of hepatocytes. (G, H) Confocal images demonstrate the sinusoidal network (green) in the liver of 1.5-month-old fish. (H) is a 2x blow-up of the area defined by the white box (G') to show the sinusoidal network and two rows of hepatocytes between two neighboring sinusoids as indicated by yellow lines. Abbreviations: ec, endothelial cells; in, intestine, L, liver; HPV, hepatic portal vein. In all images anterior is towards the left-hand side. Scale bars are 625 μm in (A-B) and 300 μm in (D-H).

After initiation of vasculogenesis, the liver grew rapidly and by 80–84 hpf it extended across the midline to the right side. Sinusoids were enlarged to 8–10 μm in diameter (Figure 3M). At 96 hpf, the liver was extended further. At 120–130 hpf, the diameter of sinusoids was around 12–16 μm, while hepatocytes of liver parenchyma were around 10–14 μm in diameter. Sequential endothelium-epithelium interaction during liver vasculogenesis significantly altered the structure of liver parenchyma. In both left and right liver lobes hepatocytes were surrounded by endothelia and almost every hepatocyte was associated with a sinusoid (Figure 3N, also see Figure 4F). Histological analysis confirmed the presence of blood cells in sinusoids at this stage (Figure 3O).

### Detection of blood flow in the embryonic liver

Although we observed an increase of sinusoids in size from the very beginning of their formation up to 120 hpf, it remained unknown when they became functional in liver. Therefore, we used a recently described non-invasive and label-free approach of fluorescence correlation spectroscopy (FCS) to determine the timing of blood flow initiation in vessels of zebrafish embryonic liver [15]. Using the LiPan/*Tg(fli1:EGFP)*^y1 ^embryos, the FCS method allowed measurement of blood flow in sinusoids of zebrafish liver *in vivo *during development. While first sinusoids were already formed in liver at around 68–70 hpf, no blood flow was detected in liver sinusoids within a depth of 70 μm from the liver surface in all analyzed larvae (n = 7, Figure 4A), but in each of them blood flow was detected in trunk vessels by FCS at this developmental stage. Soon after, at 72–75 hpf, we detected initiation of blood flow in external sinusoids of dorso-lateral part of liver parenchyma in three out of seven analyzed points (Figure 4B). It is likely that these sinusoids were already connected to the supraintestinal artery (SIA) and subintestinal veins. It seemed that the time course of initiation of blood flow in liver sinusoids correlated well with the development of intestinal vessels [8]. After initiation of the circulation, the mesh of sinusoids in the liver developed extremely fast and the liver increased significantly in size between 84–120 hpf. By 120 hpf, blood flow was detected in 8 out of 10 points located at different confocal planes as deep as 70 μm in both left and right liver lobes (Figure 4C). Due to a limit of light penetration, the detection of blood flow was restricted to 70–80 μm in depth from the liver surface. At around 120 hpf, liver vasculogenesis was almost complete and circulation was present in almost the entire liver when measured in left and right liver lobes.

Collectively, our data suggested three distinct stages of liver vasculogenesis: first, establishment of contact between ECs and hepatocytes at approximately 55–58 hpf (Figure 4D); second, formation of first sinusoids at around 58–72 hpf (Figure 4E); and third, initiation of blood flow in first sinusoids and formation of sinusoidal network (Figure 4F). At 120 hpf, confocal 3D projections revealed a dense penetrating vascular network and formation of the primary hepatic portal vein (HPV), which drained directly into the liver (18; Figure 4F).

In adult zebrafish, the pattern of sinusoidal network was comparable to that in larvae at 5–6 dpf. A confocal analysis of liver parenchyma in 1.5-mpf live LiPan/Tg(fli:*EGFP*)^y1 ^fish revealed a dense penetrating vascular network with long stretches of sinusoids separating two rows of hepatocytes (Figure 4G, H). This pattern of sinusoidal network was comparable in all liver lobes (not shown).

### The initial liver growth and organization was promoted by contact with ECs but not by blood circulation

**Figure 5:**
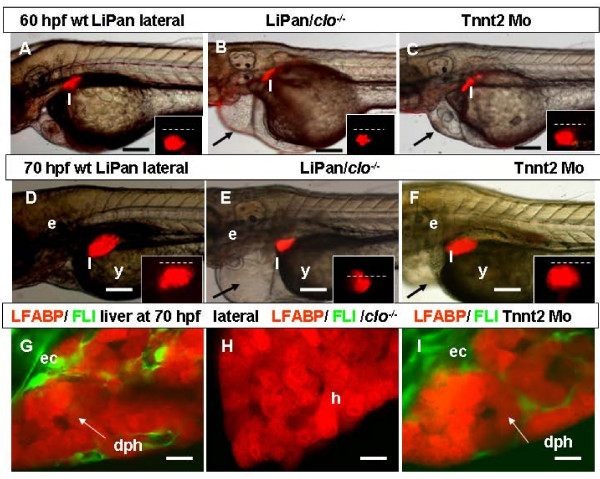
**Role of endothelia in liver development**. (A-F) Left-lateral views: liver morphogenesis in live LiPan/*Tg(fli1:EGFP)*^y1 ^larvae; wild type (A, D), LiPan/*clo*^-/- ^mutants (B, E) and LiPan/Tnnt2 morphants (C, F) at 60 hpf (A-C) and 70 hpf (D-F). Dorsal views of the liver region of the same embryos are shown as inserts in each panel and dash lines indicate the midline with the right side at the top. Note that the liver in LiPan/*clo*^-/- ^mutants is significantly reduced compared to that in controls and Tnnt2 morphants. The pericardial edemas in both clo-/- mutant and tnnt2 morphant are indicated by arrows (B, C, E, F). (G-I) Left-lateral confocal *in vivo *projections of liver of LiPan/*Tg(fli1:EGFP)*^y1 ^larvae in wild type (G), *clo*^-/- ^mutant (H) and Tnnt2 morphant (I) backgrounds. Abbreviations: bd, bile duct; dph, daisy pattern of hepatocytes; ec, endothelial cells; e, ear; h, hepatocytes; l, liver; s, sinusoid. In all images, anterior is towards the left. Scale bars, 125 μm in (A-F) and 625 μm in (G-I).

Previously, it has been shown that there are two distinct phase of liver development in the zebrafish, budding phase from 24 hpf to 50 hpf and growth phase after 50 hpf. It seems that ECs are not essential for the initiation of liver budding as the liver buds normally in *cloche *mutants that lack ECs [5]. By taking advantages of LiPan/*cloche *mutant, we confirmed that at 50 hpf the liver bud in *cloche *is similar to that in wild type siblings (not shown). The livers in LiPan/*cloche *embryos expanded normally during early growth phase; at 55 hpf, they were similar to those of controls. However, at 60 hpf, while the size of the LiPan/*cloche *and wild type embryos was comparable, the size of liver was noticeably smaller in LiPan/*cloche *embryos than that in controls (Figure 5A, B). At 68–72 hpf when the first sinusoid was formed in wild type embryos, the arrest of liver growth in LiPan/*cloche *larvae became more obvious (Figure 5D–E). Concurrently, the confocal analysis of LiPan/*Tg(fli1:EGFP)*^y1^/*cloche *did not show GFP-expressing ECs associated with hepatocytes and there was no obvious daisy pattern of hepatocyte organization (Figure 5H) as observed in controls (Figure 5G). Only in the innermost layers of *cloche *liver did we observe a few RFP-negative areas among RFP-positive hepatocytes (Figure 5H) which could represent ductal cells detected in the *cloche *mutants at 70 hpf [19]. Thus, the absence of ECs contact in *cloche *mutants might significantly affect the size of liver and this effect was even more obvious during the formation of first vessels. Apart from the reduction in size, the structural organization of hepatocytes in *cloche *liver was less obvious than that in non-*cloche *siblings (Figure 5G, H). It appears that ECs through their contact with hepatocytes provided some specific morphogenetic signals for hepatocyte organization and liver growth.

To further investigate the role of ECs in liver development, we introduced the Tnnt2 morphants, which phenocopied the zebrafish *silent heart (sih) *mutant with defects in cardiac contractility and blood circulation [20,21,2]. We found that in LiPan/Tnnt2 morphants, the liver bud formed (as detected by in situ hybridization with the *ceruloplasmin *probe, not shown) and during early growth phase was about the same size as that in controls (323/323 morphants; Figure 5A, C). A confocal examination of the liver of LiPan/*Tg(fli1:EGFP)*^y1^/Tnnt2 morphants revealed normal progression of the initial stage of EC interaction with hepatocytes at 55–58 hpf (not shown). Tnnt2 morphants suffered from a cardiac edema. Despite that increased cardiac edema was almost comparable to that of *cloche*, in contrast to the small liver in *cloche*, livers of Tnnt2 morphants (96%, 310/323) expanded notably up to 70 hpf and had about same size and shape as in controls (Figure 5D, F). Based on the examination of ~400 *cloche *mutants and > 300 morphants, we failed to see any correlation between the volume of edema and size of liver. Moreover, the livers in LiPan/*Tg(fli1:EGFP)*^y1^/Tnnt2 morphants had similar morphological organization of hepatocytes to those in non-morphant controls, in contrast to the absence of prominent daisy organization of hepatocytes in LiPan/*Tg(fli1:EGFP)*^y1^/*cloche *mutants (Figure 5G–I). Thus, it seems that in embryonic zebrafish the initial liver growth and organization is promoted by the contact with ECs but not by blood circulation,

### Importance of blood circulation for liver vasculogenesis and growth

**Figure 6:**
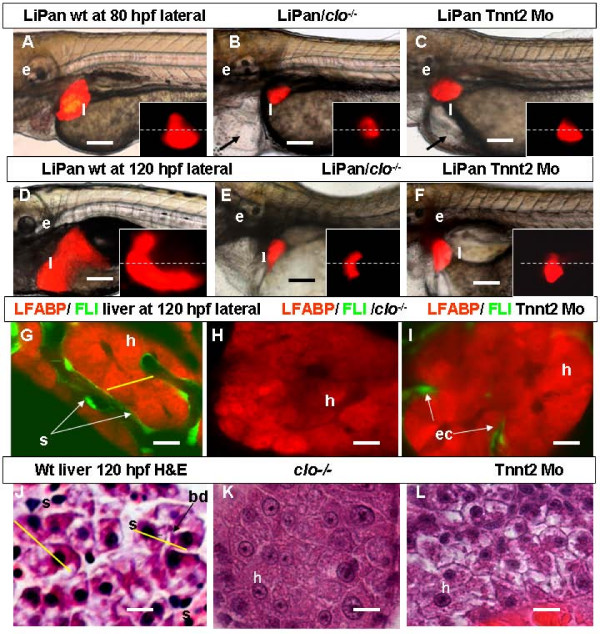
**Role of circulation in liver development**. (A-F) Left-lateral views: liver morphogenesis in live LiPan wild type (A, D), LiPan/*clo*^-/- ^mutants (B, E) and LiPan/Tnnt2 morphants (C, F) at 80 hpf (A-C) and 120 hpf (D-F). Dorsal views of the liver region of the same embryos are shown as inserts in each panel and dash lines indicate the midline with the right side at the top. Livers in *clo*^-/- ^mutants and Tnnt2 morphants are located more medial, lack the anterio-ventral and posterior expansion, and are significantly reduced in size compared to the livers in wild type sibling. The cardiac edema in both mutants and morphants is indicated by arrow. (G-I) Left-lateral confocal *in vivo *projections of liver of LiPan/*Tg(fli1:EGFP)*^y1 ^larvae at 120 hpf in wild type (G), *clo*^-/- ^mutant (H) and Tnnt2 morphant (I) backgrounds. In *clo*^-/ ^mutants ECs are absent (H), whereas in *tnnt2 *morphants they are present only between hepatocytes of the outer layer (I). (J-L) High-resolution light micrographs of hepatic parenchyma of zebrafish larvae stained with H&E. In 120-hpf wild type sibling, hepatocyte tubules are separated by sinusoids containing erythrocytes (J); in contrast, in 120-hpf *clo*^-/- ^mutant (K) and Tnnt2 morphant (L), hepatocytes are tightly connected to each other. Note two sinusoids separated by two rows of neighboring hepatocytes as defined by yellow lines. Abbreviations: ec, endothelial cells; e, ear; h, hepatocytes; l, liver; s, sinusoid. In all images, anterior is towards the left. Scale bars, 125 μm in (A-F) and 625 μm in (G-L).

After the initiation of hepatic blood circulation at 72 hpf, the liver in wild type larvae was enlarged laterally, ventrally, and across the midline. In LiPan/Tnnt2 morphants, though the liver crossed the midline and extended moderately by 75 hpf, we observed significant reduction of liver size in 95% (282/297) morphant larvae by 80 hpf (Figure 6A–C). It seemed that the liver growth in Tnnt2 morphants was arrested in late development (Figure 6D, F). The lack of blood circulation in Tnnt2 morphants eventually resulted in vascular regression. The liver phenotype of LiPan/Tnnt2 morphants progressively became similar to that of LiPan/*cloche *mutants. A confocal analysis of LiPan/*Tg(fli:EGFP)*^y1^/*cloche *mutants revealed a compact arrangement of hepatocytes similar to that observed in LiPan/*Tg(fli:EGFP)*^y1 ^siblings at the liver budding stage, i.e. hepatocytes were tightly attached to each other without obvious morphological organization (n = 8; Figure 6H). The liver of LiPan/*Tg(fli:EGFP)*^y1^/Tnnt2 morphants at the corresponding stage displayed similarly poorly organized hepatocytes with only remnants of ECs in the external layers of liver (Figure 6I) in contrast to the well developed sinusoids and organized hepatocytes in liver of LiPan/*Tg(fli:EGFP)*^y1 ^siblings (Figure 5G). Thus, by 120 hpf, the absence of circulation in Tnnt2 morphants causes liver deficiency comparable to that of *cloche *mutants lacking ECs (Figure 5G–L), indicating a vital role of blood circulation in formation of the sinusoidal network in the liver and liver growth in late development.

**Figure 7:**
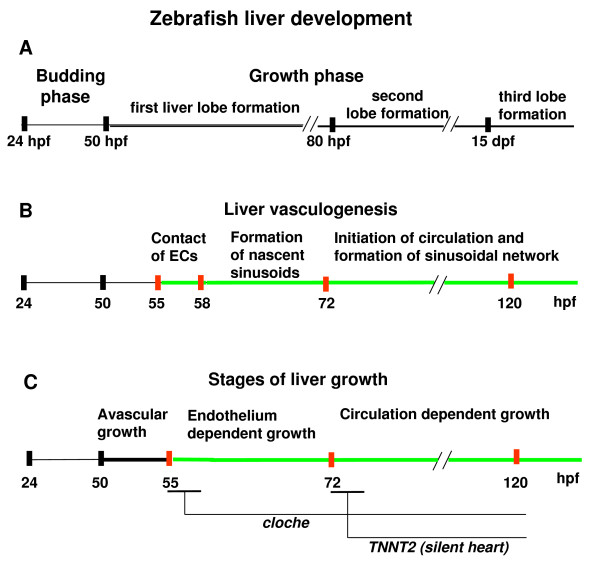
**Summary of developmental events during liver vasculogenesis and growth**. (A) Timing of liver morphogenesis in zebrafish. According to Field et al. [5], the liver morphogenesis in zebrafish consists of two phases, budding and growth. Our current study showed the formation of three liver lobs during the growth phase. (B) Timing of liver vasculogenesis. Detailed analysis of liver vasculogenesis and precise determination of blood flow initiation in liver sinusoids of live embryonic zebrafish have led us to define three phases of liver vasculogenesis: contact of ECs and hepatocytes, formation of nascent sinusoids, initiation of blood circulation and formation of the vascular network in the liver. (C) Stages of liver growth. Based on our analyses of liver vasculogenesis and growth in wild type as well as in *cloche *mutant and Tnnt2 morphant larvae, we propose three stages of liver growth: avascular growth as a transition to the growth phase, where ECs are not required; endothelium-dependent, and circulation-dependent growth stage. Approximate periods of the stages of liver vasculogenesis and growth are represented along the time line in hpf or dpf. The green time lines represent the period of liver vasculogenesis.

According to Field et al. [5] the liver development in zebrafish consists of two phases – budding and growth. Our current study further defined the timing of formation of three liver lobes during growth phase (Figure 7A). In combination with *in vivo *analysis of liver vasculogenesis and determination of initiation of blood flow in the liver of live embryos by FCS, we also defined three phases of liver vasculogenesis: 1) contact of ECs and hepatocytes, 2) formation of nascent sinusoids, 3) initiation of blood circulation and formation of the vascular network in liver (Figure 7B). Furthermore, by analysis of liver vasculogenesis and liver growth in wild type as well as in *cloche *mutant and Tnnt2 morphant larvae, we provided additional details to define three distinct stages of liver growth: 1) avascular growth, which could be considered a transition from the budding phase to the growth phase, 2) endothelium-dependent growth, and 3) circulation-dependent growth (Figure 7C).

## Discussion

### Liver morphogenesis in LiPan transgenic zebrafish

Although the early stages of endodermal organogenesis in the zebrafish have been a subject of intense studies [1-9], some of the late developmental events in liver growth and morphogenesis are still relatively poorly understood. To fill this gap, we re-evaluated liver morphogenesis in the zebrafish by focusing mainly on late events. We generated a two-color transgenic zebrafish line, LiPan, that expresses dsRed RFP specifically in the liver and GFP specifically in the exocrine pancreas to observe how liver growth is coordinated with that of pancreas during development in live embryos/larvae and adult zebrafish. The expression patterns of the two reporter genes are essentially identical to a combination of expression patterns in the two previously reported transgenic lines, *Tg(lfabp:egfp) *[16] and *Tg(elaA:egfp) *[17], both of which express only GFP in the liver and exocrine pancreas respectively. The LiPan zebrafish expresses two distinct fluorescent proteins markers with high intensity, rendering it an excellent experimental tool for detailed analysis of liver and pancreas organogenesis.

While morphological investigation of visceral organs in larvae and adult fish is often difficult due to similar colors of most of internal organs, the LiPan larvae and adults offer a convenient way to discern not only the liver and pancreas but also other internal organs due to the exclusion of the two fluorescent organs. Liver occupies the cranial region of the body cavity and the exocrine pancreas by and large occupies the posterior region of this cavity. It is clear that zebrafish, unlike some other fish, have separated and distinct liver and pancreas organs and do not form hepatopancreas (Figure 2) [23]. We consistently observed the formation of three distinct liver lobes: the first, left lobe forms at ~80 hpf; the second, right lobe at ~96 hpf, and the third, ventral lobe at 15–20 dpf during liver growth phase (Figure 1). This is in contrast to many other *Teleostei *species such as rainbow trout [24] and *Liza saliens Risso*, *Liza aurata Risso *[25], where no lobulation was recognized. In some species such as *Chondrichthyes *and *Dipnoi*, two liver lobes were found [23].

### Endothelial cells and nascent sinusoids drive liver growth and morphogenesis

Using Lipan/*Tg(fli1:EGFP)*^y1 ^transgenic zebrafish, we traced liver vasculogenesis from the very beginning until the liver became functional and showed sinusoidal network in adult zebrafish. These transgenic lines in combination with *cloche *mutant and Tnnt2 morphants, allowed us to investigate developmental mechanisms involved in liver vasculogenesis. Strong RFP expression in hepatocytes and GFP expression in ECs allowed the observation of the dynamic process of vasculogenesis in the liver from discrete ECs around the liver bud to the formation of functional sinusoids. Our study showed the dynamic formation of sinusoidal network in zebrafish liver and revealed some similarities and differences in liver vasculogenesis between zebrafish and other vertebrates.

Based upon morphological characteristics, liver development in zebrafish has been separated into two phases, budding (24–50 hpf) and growth (after 50 hpf). Hepatocytes differentiation takes place at the end of liver budding phase and before growth phase and *lfabp *is the molecular marker specific to fully differentiated hepatocytes [5,34]. It has been previously shown that ECs are adjacent to, but not encasing, the liver bud at 48 hpf [5]. In our current *in vivo *analyses, we found that during avascular growth stage, discrete ECs rim the liver bud completely and then physically interact with hepatocytes prior to blood vessels formation similar to that in mice [11]. In mouse *Vegfr2/Flk-1*^-/- ^embryos that lack mature ECs, the multi-layered liver epithelia forms but later fail to grow [11]. In zebrafish *cloche *mutant embryos, which lack ECs, the size of liver was found to be comparable to that of wild type siblings during avascular growth stage up to 55 hpf and the growth of liver was arrested during vascular stage (Figure 7B, C). It appears that the role of ECs during liver morphogenesis is conserved among vertebrates since ECs in both zebrafish and mice provide a crucial growth stimulus to the hepatic tissue before formation and function of local vessels [11]. Previous observation of liver development in the *cloche *mutant was limited to 48 hpf due to an increased severity of the cardiac edema [5]. Using live LiPan/*cloche *mutant we were able to analyze liver development at later time points. This study showed that during endothelium-dependent growth stage, the size of liver in *cloche *mutants was significantly reduced compared to that in controls and supported the previous hypothesis that the liver of *cloche *mutant may be affected during growth phase if the endothelium is important for liver development in zebrafish [5]. Furthermore, we also showed that at the cellular level, liver morphogenesis in *cloche *mutant was also affected and a prominent daisy pattern of hepatocytes was absent.

To eliminate the possibility that the arrest of liver growth in *cloche *mutants was due to the lack of blood circulation that may transport certain factors required for liver growth, we analyzed Tnnt2 morphants that lack blood circulation. In contrast to the reduced and unorganized liver in *cloche *mutants, the size of liver and the pattern of ECs and hepatocytes interaction in Tnnt2 morphants embryos were similar to that of wild type siblings during both avascular and endothelium-dependent.growth stages. Thus, ECs in zebrafish may provide some morphogenic signals important not only for liver growth but also for structural organization of hepatocytes.

Despite similarities there are some differences in liver development between zebrafish and mice. In mice, where the liver is an early hematopoietic organ, endothelial-endoderm interaction is initiated during the early phase of liver budding, whereas in zebrafish this interaction starts much later and during the growth phase. Moreover, mouse homozygous mutant *Flk-1*^-/- ^allele causes embryonic lethality by E10.5 (1 day after initiation of hepatocytes migration into the surrounding septum transversum mesenchyma) [26], but homozygous *cloche *mutants lacking ECs survive 6 days (almost 4 days after initiation of liver vasculogenesis). This time of development (5–6 dpf) corresponds with the appearance of the adult form of the heart and the transition from diffusive to convective oxygen supply.

In mice and human, ECs provide stimuli for hepatocytes to outgrow towards septum transversum mesenchyme [27,11]. Previously it has been proposed that vasculogenesis in zebrafish is achieved by endothelial invasion of liver [5]. In contrast, our data suggested that both ECs and liver grow towards each other. As ECs rim the liver completely and contact seemingly less associated surface layers of hepatocytes, the liver extends simultaneously, effectively moving hepatocytes towards endothelia. In mice, after growing into the septum transversum mesenchyme, hepatocytes organize around already formed sinusoids. In zebrafish, discrete ECs contact liver and then form nascent sinusoids. Formation of the daisy pattern of hepatocytes and nascent sinusoids proceeds at the same time. This interaction significantly changes the liver morphology, but molecular mechanisms underlying this developmental process remain to be elucidated.

### Role of functional sinusoidal network for liver growth

After the initiation of blood flow in the first liver vessels, circulation became essential for further liver growth. From 72 to 120 hpf, the liver enlarges 8–10 times in size. The developing sinusoidal network directly contributes to the increased liver volume. Before the initiation of blood circulation in the liver, the size of sinusoids is 5–8 μm in diameter, while with the blood flow in the liver, sinusoids progressively increased in size up to 12–16 μm. Before the initiation of circulation, liver size and its early structural organization depend on the presence of ECs. However, after its initiation, circulation becomes an essential factor for subsequent liver development and growth, as evident by comparative analyses of LiPan/*Tg (fli1:EGFP)*^*y*1^/Tnnt2 morphant and *LiPan*/*Tg(fli1:EGFP)*^y1^/*cloche *mutant (Figure 5J–L). Therefore, the blood circulation stimulates development of the vascular network and this network remarkably enlarges liver size because of its own volume as well as delivery of nutrients to support liver growth by cell proliferation. Further analysis of mutants with defects of vascular patterning and vessels maintenance could uncover additional critical factors involved in liver vasculogenesis [28].

The development of the hepatic vascular architecture is a multistep process through the interaction of the two tissues, hepatocytes of endodermal origin and endothelia of mesoderm origin. Further to the model on early liver development proposed by Field et al. [5], we, based on new observation of developmental events during the liver growth phase, proposed to divide the liver growth phase into three distinct stages: avascular growth as a transition to the growth phase, where ECs are not required and liver extended by proliferation of hepatocytes; endothelium-dependent growth, when liver grew due to proliferation of hepatocytes and ECs, and circulation-dependent growth stage, when the blood circulation stimulates development of the vascular network which increases liver size because of its own volume and releases nutrients to support cell growth and proliferation (Figure 7). This model could be useful as a roadmap to design further experiments addressing the role of the key factors required for liver vasculature development, the functions of signaling pathways and interactions between them during intriguing events of liver vasculogenesis.

## Conclusion

In the present study, a two-color transgenic zebrafish line (LiPan) with RFP expression in the liver and GFP expression in the exocrine pancreas was generated and the LiPan line allowed us to analyze detailed liver development in live embryos and larvae. By crossing the LiPan line with *Tg(fli1:EGFP)*^y1^, we found that liver vasculogenesis started at 55–58 hpf when ECs first surrounded the hepatocytes from the liver bud surface and then invaded the liver to form sinusoids and later the vascular network. Fluorescence correction spectroscopy detected blood circulation in the liver first at ~72 hpf. Analysis of *cloche *mutants and Tnnt2 morphants led us to conclude that both ECs and blood circulation are required for continued liver growth and morphogenesis. Collectively, we propose to divide the growth phase of liver development in zebrafish into three distinct stages: avascular growth between 50–55 hpf, where ECs are not required; endothelium-dependent growth, where ECs or sinusoids are required for liver growth from 55 hpf to 72 hpf before blood circulation in the liver sinusoids; circulation-dependent growth where the circulation is essential to maintain vascular network and to support the continued liver growth and morphogenesis after 72 hpf.

## Methods

### Zebrafish maintenance

Zebrafish were maintained in the fish facilities at the Department of Biological Sciences, National University of Singapore (NUS) and the Institute of Molecular and Cell Biology (IMCB) of Singapore according to established protocols [29] and in compliance with Institutional Animal Care and Use Committee (IACUC) guidelines. Developmental stages are presented in hour post fertilization (hpf), day post fertilization (dpf) or month post fertilization (mpf).

### Microinjection and establishment of *Tg(lfabf:ds-Red; elaA:EGFP) *zebrafish line (LiPan)

Isolation of *elaA *promoter (1.9 kb) and construction of the chimeric plasmid pElaA-EGFP have been described previously [17] The liver-specific promoter (2.8 kb) derived from the zebrafish *liver fatty acid binding protein *gene was provided by Dr. G.-M. Her and was inserted into pDsRed-Express-1 (Clontech, USA) to make the chimeric plasmid pLFABP-RFP. Both plasmids were linearized, mixed with 0.25% phenol red solution (1:1:1) at a final concentration of 100 ng/μl of each plasmid. Microinjection was carried out at the 1–2 cell stage. The DNA solution was injected into the boundary between the yolk and blastodisc. After microinjection, the embryos were maintained in egg water [29] with ~0.0005% methylene blue in a 28.5°C incubator. Transgenic founders were screened by observation of F1 embryos for RFP and GFP expression. 444 injected embryos were raised to adult and 66 of them were screened for transgenics. Two of them were found to produce F1 embryos with strong liver-specific RFP expression and exocrine pancreas-specific GFP expression. Thus, two stable transgenic lines were established and both showed standard Mendelian inheritance from F2 generation onwards. Since identical reporter gene expression patterns were observed in the two lines, only one line, named LiPan, was used for further characterization. Homozygous LiPan zebrafish were viable and had no visible phenotype. The LiPan line has been maintained in our laboratories for over eight generations and co-expression of RFP in the liver and GFP in the exocrine pancreas is always observed. Thus the two injected DNA constructs are likely co-integrated into the same chromosomal locus.

### Microscopy

To facilitate visualization of liver and exocrine pancreas of larval zebrafish in whole-mount preparations, pigmentation of skin was inhibited by raising embryos and larvae in egg water [29] containing 0.2 mM 1-phenyl-2-thiourea (Sigma, USA). Microscopic observations and photography of live embryos were performed using the dissecting fluorescent microscope SZX12 (Olympus, Japan), compound microscope Zeiss Axioscope 2 and confocal microscope Zeiss LSM510 (Zeiss, Germany). Three images were taken at the same focal plane, using a DIC filter for transmitted light for the first, epifluorescence with a Rhod for the second and FITC filter for the third. These three images were then superimposed using Zeiss AxioVision software or Photoshop (Adobe, USA). Three-dimensional confocal projections were generated using Zeiss LSM510 software (Zeiss, Germany). In all confocal studies, at each time point, 5–8 embryos/larvae from random pairs were examined.

### Blood flow detection by fluorescence correlation spectroscopy

Fluorescence correlation spectroscopy (FCS) is a single-molecule sensitive fluorescence technique which can provide information about diffusion coefficient, concentration, microfluidic flow, etc. [30,31]. It is based on an autocorrelation analysis of fluorescence fluctuations from a small focal volume in the specimen that is defined by a high numerical aperture objective and a small pinhole. Autocorrelation functions are derived for different cases such as 3D diffusion and microfluidic flow, and the models can be used to fit the experimental data. The two-flow model to extract the diastolic and systolic blood flow velocity at the same blood vessel in zebrafish larva was developed and the detailed setup was described previously [32]. The blood flow velocity in the liver sinusoids of zebrafish larvae was obtained by FCS measurement at the points of interest after the confocal image acquisition which helps to locate the position of sinusoids in the liver. In this work, each larva was anesthetized with freshly prepared 0.05–0.1 mg/ml Tricaine (ethyl m-aminoboenzoate, Sigma, Singapore) dissolved in egg water [29], immobilized in 1.5% low-melting-temperature agarose (Invitrogen, Singapore) (agarose was dissolved in 0.05 mg/ml of Tricaine in egg water), in WillCo-dish^® ^glass bottom dish (GW-3512, WillCo-Wells, The Netherlands), placed in a temperature-controlled environment and immediately proceed for measurements. For each larva, blood flow was measured in a trunk vessel as a control and then in the vessels of liver parenchyma at 7–10 randomly selected points at different depths of liver tissue up to 80 μm. For each stage 3–5 larvae were measured. With this technique, we were able to measure blood flow inside the liver parenchyma as deep as 70 μm from the liver surface.

### Generation of double (LiPan) and triple [Tg(lfabf:ds-Red; elaA:EGFP; fli1: EGFP)] transgenic cloche mutants

To visualize vascular development in the liver in live embryos, LiPan homozygotes were mated with *Tg(fli1:EGFP)*^y1 ^homozygotes and triple transgenic embryos were generated. To analyze effect of endothelia on liver development and growth, we used *cloche *mutants (*clo*^*s*5 ^[point mutation allele]) which lack almost all endothelial cells [5,12,33]. Both LiPan and LiPan/*Tg(fli1:EGFP)*^y1 ^transgenic fish were crossed with *cloche *heterozygotes to transfer the transgenes into the *cloche *mutants. After their progeny reached maturity, these fishes were crossed randomly to identify *cloche *heterozygotes that carry the transgenes. These fishes were crossed to obtain homozygous mutants with double (LiPan) or triple [LiPan/*Tg(fli1:EGFP)*^y1^] transgenic background and their development was monitored.

### Tnnt2 morphants

To analyze the role of circulatory defects in liver development, Tnnt2 antisense morpholino oligonucleotide (5'-CATGTTTGCTCTGATCTGACACGCA), which is targeted the Tnnt2 translation start codon and phenocopies the *silent heart *(*sih) *mutation [22], was obtained from Gene Tools (USA). A total of 4 ng of Tnnt2 morpholino oligonucleotide was injected into 1–2 cell stage of LiPan or LiPan/*Tg(fli1:EGFP)*^y1 ^embryos and a stable non-contractile heart phenotype was observed in 98% of injected embryos and larvae from 24 hpf to 7 dpf (their last day of survival). Morphants without obvious motility defect and lacking blood circulation (with pericardial edema) were used for analysis of liver growth and vasculogenesis.

### Histology

For cryosection, zebrafish embryos were ice-chilled and fixed with ice-cold 4% paraformaldehyde (PFA) in phosphate-buffered saline (PBS) at 4°C overnight. After fixation, embryos were embedded in 1.5% Bacto agar containing 5% sucrose and incubated in 30% sucrose at 4°C overnight. The embedded embryos were oriented and sectioned with a cryostat microtome (10 μm thickness). For paraffin sections, the embryos and larvae were fixed with either 4% PFA in PBS or Bouin's fixative, followed by paraffin embedding and sectioning (4 μm). Serial sections (from at least 3–5 embryos or larvae at each time point) were de-paraffinized, stained with hematoxylin-eosin, dehydrated, and examined.

## Authors' contributions

SK – made genetic crosses and selection, systematic analyses of organogenesis and vasculogenesis in transgenic and mutant fish, and wrote the manuscript; XP – designed and made the LiPan transgenic line; MGL – made confocal microscopic images; CLM – made genetic crosses; XP-analyzed blood flow in liver; TW- developed blood flow measurement method; VK – developed the concept of the project, wrote and approved the manuscript; ZG – developed the concept of the project, designed the LiPan transgenics, wrote and approved the manuscript.
